# Analog regulation of metabolic demand

**DOI:** 10.1186/1752-0509-5-40

**Published:** 2011-03-15

**Authors:** Nikolaus Sonnenschein, Marcel Geertz, Georgi Muskhelishvili, Marc-Thorsten Hütt

**Affiliations:** 1 School of Engineering and Science, Jacobs University Bremen, Campus Ring 1, 28759 Bremen, Germany; 2 Department of Molecular Biology, University of Geneva, Sciences III 30, quai Ernest-Ansermet, 1211 Geneva, Switzerland

## Abstract

**Background:**

The 3D structure of the chromosome of the model organism *Escherichia coli *is one key component of its gene regulatory machinery. This type of regulation mediated by topological transitions of the chromosomal DNA can be thought of as an *analog *control, complementing the *digital *control, i.e. the network of regulation mediated by dedicated transcription factors. It is known that alterations in the superhelical density of chromosomal DNA lead to a rich pattern of differential expressed genes. Using a network approach, we analyze these expression changes for wild type *E. coli *and mutants lacking nucleoid associated proteins (NAPs) from a metabolic and transcriptional regulatory network perspective.

**Results:**

We find a significantly higher correspondence between gene expression and metabolism for the wild type expression changes compared to mutants in NAPs, indicating that supercoiling induces meaningful metabolic adjustments. As soon as the underlying regulatory machinery is impeded (as for the NAP mutants), this coherence between expression changes and the metabolic network is substantially reduced. This effect is even more pronounced, when we compute a wild type metabolic flux distribution using flux balance analysis and restrict our analysis to active reactions. Furthermore, we are able to show that the regulatory control exhibited by DNA supercoiling is not mediated by the transcriptional regulatory network (TRN), as the consistency of the expression changes with the TRN logic of activation and suppression is strongly reduced in the wild type in comparison to the mutants.

**Conclusions:**

So far, the rich patterns of gene expression changes induced by alterations of the superhelical density of chromosomal DNA have been difficult to interpret. Here we characterize the effective networks formed by supercoiling-induced gene expression changes mapped onto reconstructions of *E. coli's *metabolic and transcriptional regulatory network. Our results show that DNA supercoiling coordinates gene expression with metabolism. Furthermore, this control is acting directly because we can exclude the potential role of the TRN as a mediator.

## Background

A single *Escherichia coli *chromosome comprises 4.6 Mb and must be compacted at least ~10^3 ^fold to fit inside the bacterial cell. Despite tremendous compaction the nucleoid is a dynamic structure adapted to varying rates of replication and different transcriptional requirements resulting from changes in environmental conditions. This double requirement of compaction and differential gene expression implies that bacterial chromatin must possess a high degree of spatial organization. Recent investigations indicate that the maintenance and utilization of negative supercoils in the DNA is central to both issues [1].

In the protein-free DNA molecule, DNA superhelicity is partitioned into a twist component, *Tw*, which is reflected in a twisting or untwisting of the double helix for positively and negatively supercoiled DNA respectively, and a writhe component, *Wr*, which is a measure of the three-dimensional path of the double helical axis. In a closed topological domain these quantities are related to a change in linking number (Δ*Lk*) from the relaxed state such that Δ*Lk *= Δ*Tw *+ *Wr*. Negative supercoiling can facilitate both DNA folding and compaction as well as the untwisting of DNA which is required for the initiation of transcription and replication [2].

Gene promoter regions are generally characterized by high deformability, being susceptible to duplex destabilization under conditions of superhelical stress [3-5]. The cellular promoters can be thus understood as devices channeling the free energy of negative supercoiling to localized, biologically relevant sites in DNA. Several studies using different promoters and promoter derivatives revealed that there is a distinct, yet characteristic, coupling between the superhelical density of DNA and the activity of a particular promoter [6-8]. A change of supercoiling could thus globally and differentially affect the efficiency of channeling superhelical energy at distinct promoters, allowing coordinated change of gene expression activities to occur.

**Figure 1:**
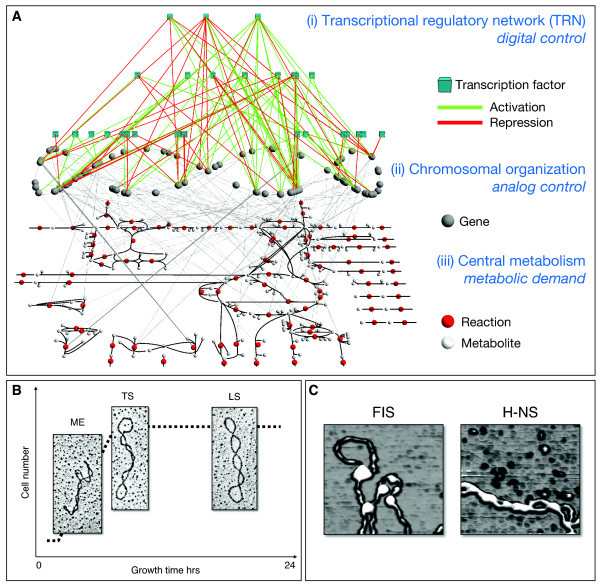
**Illustration of the different components involved in E. coli transcriptional regulation, transcription and metabolism**. (**A**) Three interconnected networks of cellular organization. Only a subset of the overall network elements is shown for the sake of clarity, i.e. only nodes and edges involved in *E. coli *central metabolism are depicted. (i) The transcriptional regulatory network (TRN): transcription factors (cyan cubes) control the expression of metabolic genes (gray spheres) either by activation (green links) or repression (red links). (ii) Chromosomal organization: DNA topology is affected globally by supercoiling (see **B**) and locally by nucleoid associated proteins (see **C**). (iii) Depiction of *E. coli *central metabolism (as described in [45]). Metabolic genes on the chromosome (ii) are connected to reactions (red spheres) according to their gene-protein/enzyme-reaction relationships. (**B**) Supercoiling energy changes across growth. The early growth phase is governed by high supercoiling, while the later phase is rather associated with low supercoiling. In addition, a wide range of environmental conditions can induce changes in supercoiling energy. In the experiments discussed here, the supercoiling energy has been altered chemically (see Methods), in order to mimic such physiological changes in a controlled fashion. (**C**) NAPs translate the global superhelical torsion into locally meaningful structures, e.g. loops (FIS) and plectonemes (H-NS).

Besides classical modes of transcriptional regulation through dedicated transcription factors (the transcriptional regulatory network), which we would like to refer to as *digital control *[9], it is well known that DNA topology affects gene expression in prokaryotes [10] as well as in eukaryotes [11], which we call *analog control *([9]; see Figure 1A).

We want to emphasize that the terminology of "digital" and "analog" control as contributions to gene regulation, which has been introduced in Marr et al. [9], is intended to emphasize the qualitative difference between regulation mediated by transcription factors and regulatory action exerted by DNA topology. We are aware that (1), when zooming into the elementary process of transcription factor diffusion and binding etc., digital control has many graded, non-binary properties and (2) stabilization of specific structural modes of the DNA by NAPs can be viewed as a discontinuous, discrete features of analog control. However, the advantage of dissecting the digital ("on or off") and analog ("more or less") logic of transcriptional regulation is in integrating the distinct types of information into a holistic approach, while separately each approach falls short of describing this multifaceted phenomenon.

In the bacterial cell the abundant nucleoid associated proteins (NAPs), including FIS, H-NS, HU, Lrp, Dps and IHF, fulfill the role of packaging and dynamic constraint of superhelicity. These NAPs are assumed to be mediators of analog control exerted by long-range nucleoprotein structures formed by binding of multiple low affinity sites in the chromosome as opposed to digital control exerted by low concentrations of dedicated transcription factors binding specific DNA sites with high affinity [9].

In particular, this combination of a global state (i.e. the superhelical density) and local states (domains and chromatin) is responsible for the spatial transcript patterns observed along the chromosome [10,12-14]. DNA supercoiling is homeostatically controlled by topoisomerase I, which leads to DNA relaxation, and DNA gyrase which introduces negative supercoils into the DNA [15]. Furthermore, the superhelical density is responsive to a range of physiological conditions, e.g. the growth phase ([16]; see also Figure 1B), phosphorylation potential of the cell [17] and stress conditions [18]. It is precisely this physiological dependence that prompted us to ask whether DNA supercoiling is a global regulator relating the chromatin structure and transcription to metabolic demand [10,19].

**Figure 2:**
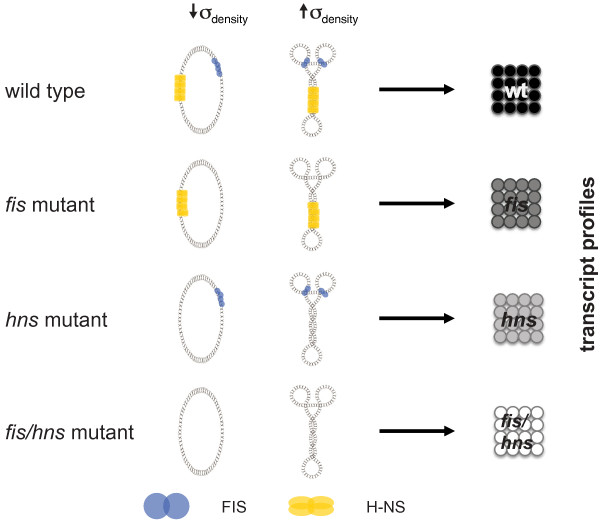
**Experimental setup**. Transcript profiles, of four *E. coli *strains (wild type, *fis *mutant, *hns *mutant and *fis*/*hns *double mutant) are compared under low (↓*σ_density_*) and high superhelical density (↑*σ_density_*), leading to four sets of differential expressed genes. The shading of the schematically depicted data sets on the right-hand side (black, dark gray, gray and white) will be used throughout the article.

In order to answer this question on a system level we utilize alterations of superhelical density, facilitated by adding the topoisomerase inhibitor norfloxacin to genetically engineered isogenic *E. coli *strains containing drug-resistant alleles of topoisomerase genes and thus selectively inhibiting either DNA gyrase or topoisomerase IV in these strains (see Methods; [10,20]) to measure supercoiling induced gene expression changes together with a combination of NAP mutations (FIS and H-NS, see Figure 1C and 2), thus precluding the buffering effects of the homeostatic network [6]. FIS is stabilizing relatively open DNA structures such as loops and interwindings readily accessible to the transcription machinery, whereas H-NS stabilizes tightly interwound plectonemic structures repressing transcription ([21], see also Figure 1c). We are here discussing the interpretational capacity of the cell: the environmental information is sensed by and filtered via chromatin structure. We show that the regulation of the metabolic state is predominantly achieved by this analog type of control.

Furthermore, we show that the regulatory control exhibited by DNA supercoiling is not mediated by the transcriptional regulatory network (TRN), as the consistency of the expression changes with the TRN logic of activation and suppression is strongly reduced in the wild type in comparison to the mutants. Our data are evidence for an optimal conversion of supercoiling into metabolic adjustments by NAPs.

While it is true for eukaryotes that the multi-level organization of gene regulation obfuscates the connection between mRNA and protein levels, let alone metabolic fluxes, and it seems that most of the control on metabolism is contributed by the post-transcriptional levels [22], the situation is known to be quite different in prokaryotes where transcription and translation are tightly coupled [23,24]. So it is valid to analyze the role of transcriptional regulation in order to understand bacterial homeostasis and the metabolic state of a cell.

To our knowledge, this work is first to show directly on a system-wide level the coordinated regulation of cellular metabolism by DNA supercoiling and NAPs.

## Results and Discussion

### Analysis strategy

An important feature of our approach is that we analyze subnetworks of the overall metabolic gene network defined by the data at hand. These *effective networks *contain only the active components (differentially expressed genes) under the given conditions (alterations in the superhelical density) and are analyzed from a network-topological perspective. The connectivity of these gene-centric effective networks is thus a result of the underlying reaction-centric topology, together with the observed gene expression pattern. Deviation of this connectivity from randomness is what we will in the following call *metabolic coherence *(*MC*). A second, more refined definition of effective metabolic gene networks, which will also be used in the following, requires both a significant expression change for one of the associated genes and a non-zero metabolic flux predicted for the encoded reaction using flux balance analysis [25] under specified environmental conditions.

**Figure 3:**
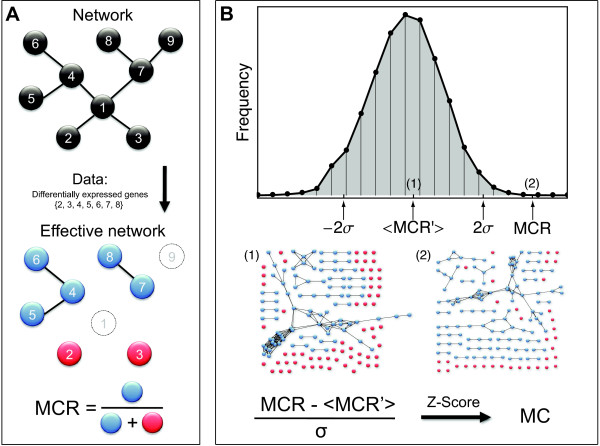
**Effective gene networks and metabolic coherence**. (**A**) Scheme depicting the calculation of the metabolic coherence ratio *MCR *for afictitious network and data set. The data (genes with significantly changed expression) are mapped onto the network resulting in an effective subnetwork. The metabolic control ratio is then the ratio of connected nodes (blue) and all nodes, i.e. the sum of connected and isolated (red) nodes, in the effective network. Unhighlighted nodes correspond to genes with no significant expression changes. (**B**) Calculation of the metabolic coherence. Randomly reselecting the same number of affected nodes in the network allows the sampling of random effective networks and thus the computation of a set of random metabolic control ratios *MCR'*. These allow the computation of a z-score value termed metabolic coherence *MC *for the MCR. (2) is an example of a real effective network, whereas (1) is one of its random counterparts. *MCR' *of (1) lies approximately around the mean <*MCR' * >.

The coherence of metabolism and gene expression patterns is quantified as follows (details are given in Methods and Additional file 1: Supplemental Text S1): we map the patterns of differentially expressed genes, i.e. genes that are responsive to a variation of the negative supercoiling (see Figure 2), from the four genetic backgrounds (wild type; *fis, hns, fis/hns *double mutants, respectively) directly onto a metabolic gene network in order to extract effective networks. Then we compute the ratio of connected nodes and all nodes in the effective network, which we call *metabolic coherence ratio *(*MCR*). This quantity is then converted into a z-score, by using a random distribution of expression changes as a null model (Figure 3 summarizes this procedure), which is our *metabolic coherence *(*MC*) in the following. The *MC *allows us to compare the amount of network coherence between gene expression profiles and metabolic pathways for the different data listed in Figure 2.

In order to validate our results on a broad scale we use network reconstructions from multiple independent databases and also apply different methods to handle gene-reaction mappings as well as currency metabolites (see also Methods and Additional file 1: Supplemental Text S1). In the following we will present our results for the different variants of the metabolic coherence for the four gene expression profiles from Figure 2.

### Metabolic coherence

**Figure 4:**
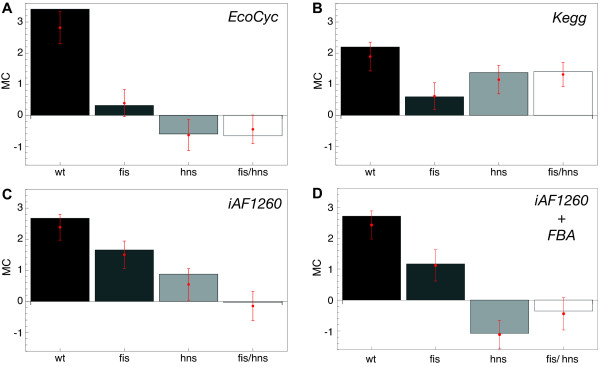
**MC for four independent E. coli metabolic network reconstructions**. (**A**) The network obtained from the EcoCyc database pathway information. (**B**) The network obtained from the KEGG pathways. (**C**) The network subset of the *i*AF1260 network where currency metabolites have been removed manually. (**D**) The *i*AF1260 network (currency metabolites have been removed manually) consisting only of reactions active under a rich medium condition. Error bars represent the standard deviation of a jackknife test, where the MC was recomputed 100 times by discarding 10% of the transcript data for each of the four genetic backgrounds.

In Figure 4 the four values of the *MC *(for the wild type and the three mutants) are shown for three different metabolic network representations, namely for the EcoCyc database [26], for the KEGG database [27] and for the *i*AF1260 metabolic model [28].

Figure 4A displays the pattern retrieved from the gene network based on the EcoCyc pathways. The wild type expression data exhibit the strongest coherence with the metabolic network (high *MC*). The wild type also shows the strongest *MC *for the KEGG network compared to the three mutants, however less clearly than for the EcoCyc case (see Figure 4B). Figure 4C gives the *MC *pattern for the *i*AF1260 gene network, from which we manually removed currency metabolites. In this *in silico *model of *E. coli *metabolism, we again observe a strong *MC *for the wild type and low values for the mutants, with the double mutant exhibiting the lowest amount of coherence.

Flux balance analysis (FBA) is a quantitative approach for computing steady-state fluxes on metabolic networks [23]. It allows us to study whether the observed systematics are enhanced, when only active links in the metabolic network are taken into account. Using the *i*AF1260 model, we computed a steady-state flux distribution that maximizes biomass production [28] under a rich medium condition and eliminated all inactive links from the network. The resulting *MC *is shown in Figure 4D. Strikingly, the restriction to active fluxes enhances the previous pattern (from Figure 4C), i.e. the gap between the wild type metabolic coherence and the *MC *values of the three mutants.

The key observation from Figure 4 so far is that changes in gene expression levels brought about by changes in supercoiling energy in the genome have a strong metabolic interpretation: the agreement of these expression changes with the metabolic network is significantly above randomness (as measured by the metabolic coherence). When severely perturbing the internal mechanisms of chromatin organization (by eliminating FIS and/or H-NS from the system), metabolic coherence goes down.

### Robustness of the result

Network analysis has established itself as an efficient way of exploring biological systems ([9,29]; see also Additional file 1: Supplemental Text S1). Nevertheless, network treatment of metabolic systems is accompanied by certain difficulties and we check the robustness of our results against many of them. In order to solidify this initial result, we need to look in detail at several issues, which can potentially affect our analysis (see also Additional file 1: Supplemental Text S1):

(*i*) Gene to reaction mapping. While all our analyses have been performed with gene-centric graphs, the reaction-centric graph serves as the starting point for assessing metabolic information (in particular, the activity of metabolic fluxes). Decisions are therefore necessary, how to relate the reaction level with the gene level. The procedure of mapping genes (i.e. the layer of information, where expression changes occur) onto reactions (i.e. the layer of information, where the metabolic network is evaluated) can have an impact on our result. Excluding ambiguous gene to reaction relations in a step-wise fashion permits us to investigate if our results are sensitive to this issue.

(*ii*) Treatment of currency metabolites. Currency metabolites are compounds in metabolic reactions balancing charge, energy, phosphate etc. They are distinguished from main metabolites (which define the metabolic pathway structures) only by biochemical knowledge or, qualitatively and indirectly, due to their very high degree in the metabolic network (resulting from their involvement in a vast number of reactions). The treatment of currency metabolites is an important issue in the discussion of the topological properties of metabolic networks (see, e.g., [30]). An approximate way of eliminating currency metabolites from metabolic network representations is to remove a certain percentage of highest-degree metabolites. Alternatively, one can use a database, where metabolites are already labeled as main metabolites and currency metabolites, respectively. This information is included in the most recent variants of the KEGG database (e.g., release 51.0; see [27]). In the *E. coli *FBA model *i*AF1260 [28], this information is not available. In order to obtain a currency metabolite free version of *i*AF1260 we used either a threshold to remove 4% of the most highly connected metabolites (*threshold heuristic*; comparable to the procedure described in [31]) or a manually curated network (resembling the procedure described in [30]; see also Additional file 1: Supplemental Text S1).

(*iii*) Differences between metabolic databases. Using intersections of the different metabolic reconstructions of *E. coli *allows us to focus on the commonalities between them.

(*iv*) Definition of the growth medium for determining the active metabolic reactions via FBA. All these points are addressed in the following.

### Large-scale evaluation

**Figure 5:**
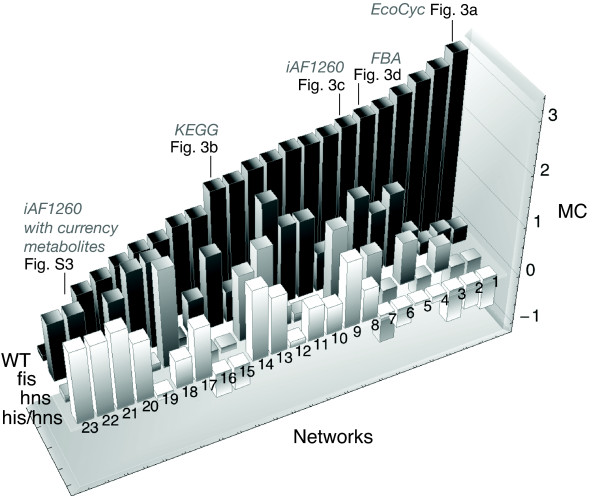
**Results for the MC analysis for all available network reconstructions sorted by the size of the wild type MC**. Notation: network* - linked reactions with an overlap in the underlying gene set have been omitted; network** - only linked reactions are included, where both are associated with single non-overlapping genes; iAF1260*_man _*- currency metabolites have been removed manually; iAF1260*_deg _*- currency metabolites have been removed by degree threshold; iAF1260 - the untreated network (KEGG and EcoCyc are per construction free of currency metabolites); in the following (*k*) denotes slice number *k *in the chart. (1) EcoCyc, (2) EcoCyc*, (3) Intersection of EcoCyc and KEGG networks, (4) Intersection of EcoCyc and iAF1260*_man_*, (5) , (6) iAF1260*_man _*obtained from FBA (rich medium), (7) iAF1260*_man_*, (8) EcoCyc**, (9) KEGG*, (10) iAF1260*, (11) iAF1260*_deg_*, (12) , (13) Flux-coupling network (fully coupled), (14) KEGG, (15) Intersection of KEGG and iAF1260*_man_*, (16) Intersection of EcoCyc, KEGG and iAF1260*_man_*, (17) KEGG**, (18) Flux-coupling network (fully and directionally coupled), (19) , (20) iAF1260**, (21) Flux-coupling network (directionally coupled), (22) iAF1260, (23) iAF1260*.

Figure 5 shows the *MC *signatures (sorted by size of the wild type *MC*) for a large compendium of metabolic gene networks. These networks can be subdivided into five categories (*MC *values for all networks and data sets shown in Figure 5 can be found in Additional file 1: Supplemental Table S2): (*i*) The most basic setup are metabolic gene networks extracted from the EcoCyc, KEGG and *i*AF1260 database.

(*ii*) In order to evaluate the influence of the gene-reaction mapping on our results, we computed *MC *values for all databases using the following configuration: (a) Taking all multiplicities into account, (b) excluding cases where a single or multiple genes are associated with two consecutive reactions and (c) taking only reaction links (pairs of reactions sharing a metabolite) into account, which are associated with two single distinct genes (see also Additional file 1: Supplemental Text S1 and Supplemental Figure S2).

(*iii*) We also computed signatures for different intersections of all available databases. By doing so, we gradually remove uncertain connections between genes, nomenclature issues and differences in the level of chemical detail captured by the different databases. This increases the confidence of the used gene network. The intersection of the gene networks from KEGG, EcoCyc and the *i*AF1260 model constitutes hereby the network with the highest confidence as it includes only connections being present in all databases. It should be noted that the differences in the results under variation of the database are also due to the balance between enhancing the systematic contribution (e.g., by eliminating currency metabolites) and retaining a large enough network to extract statistically meaningful quantities.

(*iv*) Different treatments of currency metabolites in case of the *i*AF1260 network (see Additional file 1: Supplemental Text S1 and Supplemental Figure S3 and S4): (a) manual curation, (b) threshold heuristic and (c) no treatment.

(*v*) Recently, flux-coupling networks have been intensely studied in terms of their organizing principles and their relation to gene expression data. A flux-coupling gene network coming from [32], which has been obtained from the *i*JR904 *E. coli model *[33], is analyzed here. It is subdivided into three subsets: (a) The total network, and two subsets, i.e. (b) fully and (c) directionally coupled gene pairs.

The overall trend seen in Figure 5 is that metabolic coherence is highest in the wild type. The mutants' expression patterns, while displaying a positive *MC*, are not as well aligned to the metabolic network as the wild type. This effect is particularly clear when only switched-on fluxes are taken into account. In this case the metabolic coherence directly measures the coherence of the expression pattern with the pattern of metabolic fluxes. Furthermore, we find a similar pattern for the fully-coupled flux-coupling gene network, which indicates that besides the topological matching also other metabolic relationships are perturbed in the mutants.

Qualitatively speaking, considering intersections and restricting the analysis to fluxes, which are predicted active by FBA, enhances the dominant signal of high wild type metabolic coherence compared to the mutants.

### Growth medium complexity

**Figure 6:**
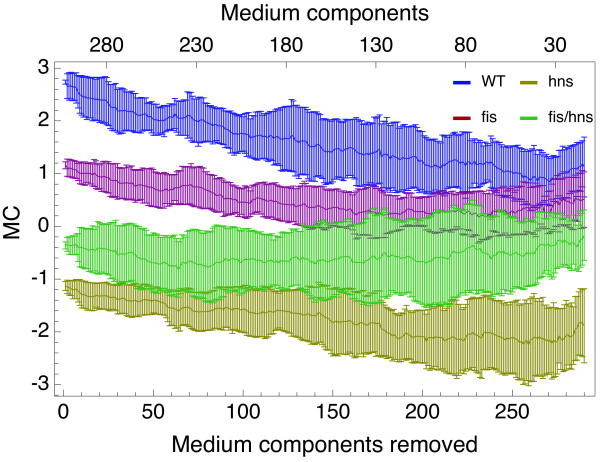
**MC under varying media conditions**. Starting from a rich medium, medium components are removed one by one under the condition that biomass production is not disrupted until a minimal medium composition is reached. Mean MC values over 20 simulations are shown for the wild type (blue), *fis *(red), *hns *(yellow), and *fis*/*hns *double mutant (green) effective gene network. Error bars represent the standard deviation.

In order to assess the robustness of the result obtained from the flux-activity network shown in Figure 4 D, it is instructive to analyze how the metabolic coherence (and in particular the strong differences between wild type and mutants) depend on the growth medium: for Figure 6 we start out with a rich medium and iteratively remove components until we reach a minimal growth medium. Thus the starting points of the four *MC *curves in Figure 6 coincide with the *MC *values shown in Figure 4D. When going from a rich to a minimal medium, the number of active genes increases (see Additional file 1: Supplemental Figure S1), as more and more reactions have to be switched on to compensate for the decreasing nutrient availability. Additionally, from left to right we are deviating ever more strongly from the experimental conditions behind the gene expression data. The main result in Figure 6 is that the clear separation of the wild type metabolic coherence from the mutants' persists over a wide range in medium complexity. Furthermore, when approaching a minimal growth medium, discrimination of *MC*s is strongly reduced.

### Link to digital control

Is the strong metabolic coherence found for wild type *E. coli *a direct consequence of chromatin organization (analog control) or is it mediated indirectly through the transcriptional regulatory network (TRN)? From [9] we know that digital control (i.e. the consistency of the analyzed gene expression patterns with the TRN) is low in the wild type (compared to the FIS and H-NS mutants) on the network-wide scale, indicating a buffering effect of the TRN. In this study [9], digital control has been measured using the *digital CTC *(Control Type Confidence), a measure very similar to our metabolic coherence (see Methods), that evaluates the coherence of patterns of differentially expressed genes with the TRN.

**Figure 7:**
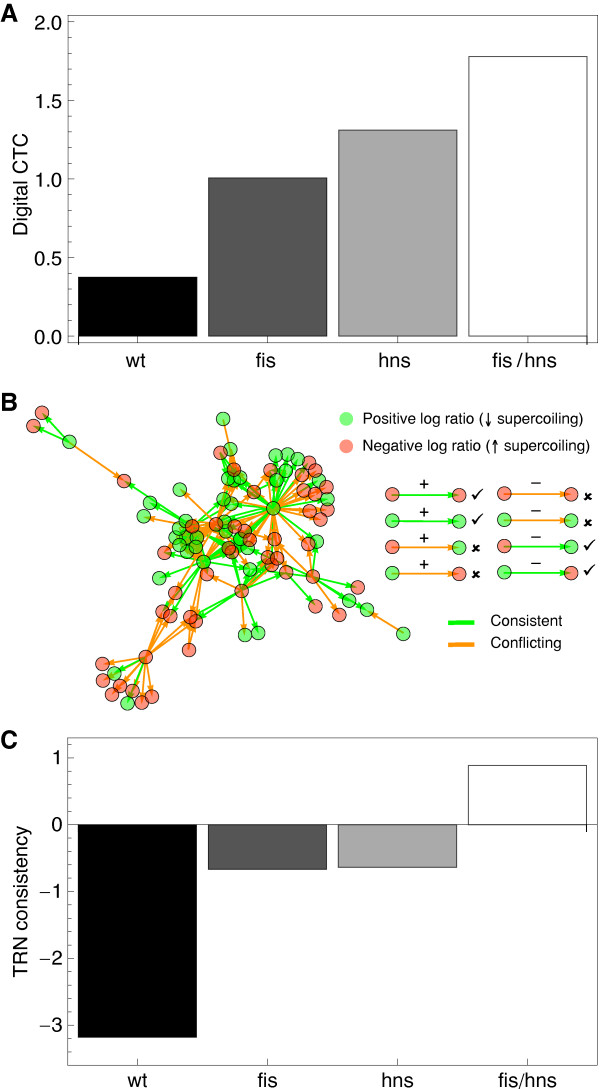
**TRN consistency**. (**A**) Digital CTC (digital control) for the four genetic backgrounds. (**B**) The effective TRN (including only metabolic genes and their regulators) for the double mutant data (*fis*/*hns*). The scheme on the right-hand side explains the classification of consistent (checkmark; green link color) and inconsistent (x; orange link color) links. (**C**) Consistency of the signs of supercoiling-induced gene expression changes with the transcriptional regulatory network.

Here we measure the digital CTC for a part of the TRN that only consists of regulatory actions (links) between metabolic genes found in the EcoCyc network and genes coding for transcription factors. As expected, the digital control measured as the *digital CTC *([9]) is significantly lower in the wild type in comparison to the mutants (see Figure 7A).

Beyond the standard digital control strength from [9] we also integrate the signs of the expression changes with the regulatory information on the corresponding links in the TRN (see Figure 7B and Methods). This is an elegant method for strengthening the direct link between supercoiling and the metabolic network: not only is the pattern of supercoiling-induced gene expression changes meaningfully distributed on the metabolic network, but also the transcriptional regulatory network does not provide an adequate interpretation of the data (see Figure 7).

## Conclusions

Changes to the superhelicity of the bacterial chromosome cause patterns of gene expression changes [14], which have been discussed from a signal processing point of view [13], a global perspective (including an enrichment analysis of metabolic pathways) [10], and in the context of transcriptional regulatory and spatial gene-proximity networks (spatial proximity on the genome) [9]. Our main result, the high metabolic coherence of supercoiling-induced gene expression changes in wild type *E. coli*, as opposed to mutants lacking the NAPs FIS and H-NS, provides further evidence for a regulatory role of DNA supercoiling. It is robust across several metabolic databases and over a wide range of environmental conditions, when taking flux-activity predictions into account. Furthermore, it is not qualitatively affected by technical details of defining the metabolic network. We can only bring these *MC *values down by mutations perturbing the machinery of chromosomal organization. These mutants are still viable, but their pattern of supercoiling-induced gene expression changes shows a markedly reduced metabolic coherence. They are, in fact, close to random expression changes, suggesting that the altered overall superhelical density and topological barriers in these mutants [10,12] preclude efficient channeling of the changes of superhelicity into metabolism.

Furthermore, the low consistency of the wild type expression patterns with the TRN topology (digital control) and its encoded regulatory logic (TRN consistency), suggest that the transcriptional regulation of enzymatic genes is primarily accomplished by chromosomal organization, i.e. the concerted interplay of global supercoiling and NAPs. Quite contrary, the stronger consistency of mutant expression changes and TRN topology and logic support the view in [9] that the TRN is buffering the lack of NAPs.

The results presented here, while providing a fairly clear picture of the interplay between mechanisms of gene regulation and metabolism, provide several incentives for our analysis as obvious steps for future work: at the core of our analysis is the metabolic coherence. It would be helpful to compare this measure with related attempts of quantitatively comparing gene expression data with metabolic information [34]. Also, if suitable data are available, we would like to extend our analysis to other organisms. A more careful discussion of the gene-reaction mapping from a network perspective is certainly necessary in order to go from our observation of metabolic coherence to a more detailed interpretation. It also may be helpful to manually construct metabolite, reaction and gene mappings between *i*AF1260, KEGG and EcoCyc, in order to better understand the strong differences in *MC *between the databases.

On a broader level, we believe that the general approach of defining and comparing control strengths and topological coherence measures associated with distinct biological processes and, in this way, dissecting gene expression patterns, may be a useful perspective for systems biology investigation, where a multitude of influences shape a process at hand. In those cases where the control type under investigation is network-based (like the metabolic coherence defined here), control strength evaluates effective networks (defined as the currently active part of the static background network). Such effective networks are a novel and highly instructive way of exploring the relation between network architecture and dynamical processes (see, e.g. [35], for an analysis of effective gene regulatory networks and [36], for a theoretical study of effective networks).

## Methods

A detailed description of materials and methods is given in Additional file 1: Supplemental Text S1.

### Gene-centric metabolic networks

We represented metabolism in form of a connectivity network of metabolic genes. We define metabolic genes *G *as DNA units that encode enzymes or parts of enzyme complexes. Let the gene product of gene *G*1 be involved in reaction *R*1 and that of *G*2 in *R*2. In the gene-centric metabolic network we study here, the two genes *G*1 and *G*2 are directionally connected if and only if the same metabolite exists among the products of *R*1 and the substrates of *R*2. Networks representing the full metabolism of *E. coli *K12 MG1255 have been constructed from the following sources: the EcoCyc [26] pathways were extracted from the pathways.dat file contained in the flat-file distribution (EcoCyc version 13.6). Neither signaling nor superpathways have been considered in our analysis. The KEGG pathways [27] were retrieved from a distribution of xml files (ftp://ftp.genome.jp:21/pub/kegg/xml/organisms/eco/; extracted on 20 November, 2009) describing the different pathways included in the KEGG database. The *in silico *reconstruction *i*AF1260 [28] was obtained in SBML format [37] from the BIGG database [38]. In order to avoid irrelevant connections coming about due to highly abundant compounds, e.g. ATP or other cofactors, sometimes termed currency metabolites [30], we utilized data sources (EcoCyc, KEGG) where these metabolites already have been removed on a reaction to reaction basis. In lack of this information (like in *i*AF1260) we employed a threshold on the metabolites' connectivity degrees to exclude those factors prior to network construction or removed them manually (see also Additional file 1: Supplemental Text S1 and Supplemental Figure S4).

### Metabolic coherence

For each effective subnetwork ***N ***the ratio of connected nodes to overall nodes was calculated as the metabolic coherence ratio *MCR*. To make this measure robust against sample size effects we transformed it into a z-score, the metabolic coherence *MC*, by mapping random gene sets of the same size (i.e. the number of genes/nodes in the effective subnetwork *G*) onto the overall static network, thus constructing random effective networks ***N' ***with associated *MCR' *values. The *MC *was computed using 5000 realizations of the null model. A jackknife test was sometimes used to verify the robustness of the *MC*. The *MC *was recalculated 100 times while randomly removing 10% of the expression data.

### TRN consistency

The *E. coli *transcriptional regulatory network was obtained from RegulonDB [39] (version 6.4). Only links between transcription factors (regulators) and metabolic genes (as found in the EcoCyc network) were considered for the digital control and TRN consistency analysis. Digital control was measured in form of the *digital CTC*, as described in [9], with the exception that the ratio of connected nodes to overall nodes was used instead of the ratio of connected to isolated nodes. Methodologically, this method is similar to the *MC *computation. The consistency of effective TRN subnetworks (TRN consistency) was calculated as the ratio of consistent links (i.e. the regulatory logic encoded on the links is consistent with the expression signs on the nodes; see Figure 7B) to overall effective links. Similar to the *MC*, this ratio was transformed into a z-score. Shuffling the expression signs of the effective nodes was used as a suitable null model. 5000 realizations of the null model were used for the z-score transformation.

### Constraint-based modeling

Constraint-based models [40] and especially flux balance analysis (FBA; [25]) and its variants allow the prediction of steady-state flux distributions for genome-scale metabolic models by solving a linear optimization problem under various subsidiary conditions. This approach has been used thoroughly in the past to tackle a wealth of questions regarding the metabolic capabilities of different organisms [40,41]. For the computation of flux distributions under varying media conditions, we started from a rich medium, removing medium components one by one under the condition that biomass production is not disrupted until a minimal medium composition was reached. We should mention that a large number of trajectories through the traversed media space exists.

### Experimental setup

The transcript profiles analyzed in this study were obtained by DNA microarray analyses using genetically engineered *E. coli *LZ41 and LZ54 strains containing norfloxacin-resistant topoisomerase gene alleles to selectively inhibit either DNA gyrase or topoisomerase IV activity and respectively induce either relaxation or high negative supercoiling [20].

The generation of the *fis *and *hns *single mutants of *E. coli *LZ41 and LZ54 strains, their growth and treatment conditions are described in [10]. The *fis *and *hns *double mutants were generated by P1 transduction of mutant alleles from donor strains into the *E. coli *LZ41 and LZ54 strains used in previous study for investigation of the effects of single mutations [10]. The strains were grown in 2 × YT medium at 30°C. Total RNA isolated from exponentially growing LZ41Δ*fis*Δ*hns *and LZ54Δ*fis*Δ*hns *strains after brief (15 min) treatment by norfloxacin was subjected to DNA microarray-mediated transcription profiling using OciChip *E. coli *K12 V2 Arrays according to OciChipTM-Application Guide (http://www.ocimumbio.com) as described in [10].

Introduction of the *fis *and *hns *mutations in the LZ41 and LZ54 strains did not alter the global supercoiling response to drug addition [10]. By adding norfloxacin to the LZ41 and LZ54 strains and their mutant derivatives we could vary the superhelical density *σ_density _*in opposite directions and distinguish gene transcripts associated either with relaxation (↓*σ_density_*) or high negative supercoiling (↑*σ_density_*) in each genetic background.

In brief, for each comparison two biological replicates with two technical replicates were performed, resulting in a total of 8 hybridizations. Scanned array images were analyzed using the TM4 software package [42]. Spot intensities were quantified and the quality of each spot was verified by calculating a quality control (QC) score depending on signal-to-noise ratio for every channel and calculating p-values for each channel (as result of a t-test comparing the spot pixel set and surrounding background pixel set) using the TIGR Spotfinder software. Data was normalized by locally weighted linear regression [43]. A one-class t-test [44] was applied to obtain differentially expressed genes within each data set (significance level *α *< 0.05). The microarray data has been deposited to the ArrayExpress database (ArrayExpress accession numbers: E-MEXP-462, E-MEXP-463, E-MEXP-3049, and E-MEXP-3050.)

## Authors' contributions

NS, MG, GM and MTH designed research. NS, MG performed research. NS, MG analyzed data. NS, GM and MTH wrote the paper. All authors read and approved the final manuscript.

## Supplementary Material

Additional file 1 **Supplements**. Extended methods section (Text S1), including supplementary Figures S1-4 and Table S1. Click here for file
