# Supplementary material for: Analog regulation of metabolic demand
Source: BMC Syst Biol. 2011 Mar 15;5:40. doi: 10.1186/1752-0509-5-40 (PMC3068955; doi:10.1186/1752-0509-5-40)
Supplement: Additional file 1 — Supplements. Extended methods section (Text S1), including supplementary Figures S1-4 and Table S1. [file 1752-0509-5-40-S1.PDF]

Text S1 (Analog regulation of metabolic demand)

Nikolaus Sonnenschein[1,*], Marcel Geertz[2], Georgi Muskhelishvili[1], Marc-Thorsten Hütt[1]
1 School of Engineering and Science, Jacobs University Bremen, Bremen, Germany
2 Department of Molecular Biology, University of Geneva, Geneva, Switzerland
∗ E-mail: n.sonnenschein@jacobs-university.de

Metabolic network representations

The strength of graph theory is that it can represent a complex system in a unified formal language of nodes and links. Examples of graph-theoretical analyses of metabolic systems are [1] and [2]. Essentially, the pattern of zero and non-zero entries of the stoichiometric matrix defines a graph representation of the metabolic system. The level of information conveyed by looking at metabolism from a graph-theoretical perspective is still subject to constant scrutiny [3].

A suitable approach for analyzing the correspondence between expression changes and metabolism and thus quantifying metabolic coherence is the application of the tools developed for the effective TRNs [4] to a gene-centric representation of metabolism, where the nodes are metabolic genes and a link is drawn between two genes, if the associated reactions share a common metabolite [5]. This representation is the gene-centric variant of one of the standard projections of a bi-partite graph representation of metabolism, where both, metabolites and reactions serve as node sets (see, e.g., 6, for a discussion of these and other representations).

Additional forms of representation include metabolite-, reaction-, enzyme- or gene-centric views. The metabolite-centric view represents the interconversion possibilities of the different substrates, whereas other views concentrate more on the processes (reactions), protein (enzymes) and genomic (genes) levels respectively. We chose the gene-centric view for our analysis as it allowed us a direct comparison of expression patterns with metabolic pathways.

Reaction to gene mappings

Imagine the following scenario: a reaction is catalyzed by a single enzyme (no isozymes involved), encoded by a unique gene (no enzyme complexes involved). With this simple scheme in mind one might conclude that reaction-, enzyme- and gene-centric representations are redundant. However, most of the time reactions and their associated enzymes and genes are not interchangeable. Figure S2 visualizes the amount of multiplicities between the reaction- and gene-level. The columns of the colored grid represent the absolute number of genes per reaction pair. The rows represent the number of unique genes. So the number of consecutive reaction pairs sharing a single gene can be found in column 2, row 1, as both reactions are associated with a single gene and it happens that it is the same for both. In contrast, the previously described simple scheme can be found in column 2, row 2. In order to assess the impact of these ambiguities on our results we constructed other graphs in addition to our gene-networks by applying the following rules: (1) the removal of reaction pairs lying beside the diagonal of the grid (see Figure S2) excludes situations where a single or multiple genes are involved with both reactions; (2) taking into account only reaction pairs fulfilling the condition of the second column and row, thus effectively excluding enzyme complexes and the situations described in (1).

Currency metabolites

Another problem emerges through highly connected compounds, which have been termed current or currency metabolites in the past [7, 8]. They have caused reports of questionable average path lengths

[2, 9] as they represent unrealistic shortcuts obscuring the essential pathway structures that have been assembled by biochemists over the last century.

For example Figure S3 demonstrates the huge impact of currency metabolites on our MC results by showing the z-score pattern for the untreated iAF1260 network. All scores are basically below or a little above 1 and such no significant coherence could be measured.

However, the KEGG and EcoCyc data sets provide currency metabolite free representations through their human readable pathway maps (in contrast to their complete reaction databases). For iAF1260 [10] we employed on the one hand a threshold heuristic to remove a certain percentage (i.e. 4 % for the results shown in the main article) of the most highly connected metabolites as described by Kharchenko et al. [5], and on the other hand a manual curation of the network where currency metabolites were removed on a reaction to reaction basis, i.e. the approach described by Ma and Zeng [8]. Figure 3C (in the main text) shows the MC result for the manually curated network for comparison with the untreated one (see Figure S3). Figure S4 shows the dependency of the MC on the percentage threshold.

Constraint-based modeling

For a metabolic system consisting of N reactions and M compounds the linear programming (LP) formulation of FBA can generally be stated as follows:

$$\begin{array}{lll} \text{Maximize} & Z = \mathbf{c} \cdot \mathbf{v} & \\ \text{subject to} & \sum_{j=1}^{N} S_{ij} v_j, & i = 1, \ldots, M \\ & v_j^{min} < v_j < v_j^{max}, & j = 1, \ldots, N \\ & v_j^{(m,s)} < v_j^{(t)} < v_j^{(m,u)}, & j = 1, \ldots, N^{(t)} , \end{array} \tag{1}$$

where v is a vector of reaction fluxes constrained by the stated boundary conditions, S is a matrix storing the stoichiometric information of the system (i.e. S_{ij} is the stoichiometric coefficient of metabolite i in reaction j) and Z denotes the objective to be maximized represented by a linear combination of fluxes v_j and objective coefficients c_j. Here, $v^{(t)}$ denotes a transport reaction, i.e. a reaction either secreting metabolites from the system or taking them up. The quantity $N^{(t)}$ is the number of transport reactions. As an approximation to a rich medium condition we allowed for every available transport reaction $v^{(t)}$ unlimited secretion $v^{(m,s)} = -\infty$ and $v^{(m,u)} = 20$ [in units of $mmol/g \cdot dw \cdot h$] as an arbitrary upper bound to influx. With the exception of $v^{(t)}$, all reversible reactions were treated as two distinct irreversible reactions. Maximization of biomass production [10] and simultaneous minimization of all other fluxes was used as Z in order to avoid accumulation of flux in cycles. As all constraints are linear and the solution space is convex, a global maximum can always be found using linear programming (assuming the problem is well defined and not unbounded), though multiple global optima cannot be excluded [11].

References

1. Guimerà R, Amaral LN: **Functional cartography of complex metabolic networks**. *Nature* 2005, **433**(7028):895–900.

2. Jeong H, Tombor B, Albert R, Oltvai ZN, Barabási AL: **The large-scale organization of metabolic networks**. *Nature* 2000, **407**(6804):651–654.

3. Montañez R, Medina MA, Solé RV, Rodríguez-Caso C: **When metabolism meets topology: Reconciling metabolite and reaction networks**. *BioEssays : news and reviews in molecular, cellular and developmental biology* 2010, **32**(3):246–56.

4. Marr C, Geertz M, Hütt MT, Muskhelishvili G: **Dissecting the logical types of network control in gene expression profiles**. *BMC Syst Biol* 2008, **2**:18.

5. Kharchenko P, Church GM, Vitkup D: **Expression dynamics of a cellular metabolic network**. *Molecular Systems Biology* 2005, **1**:2005.0016.

6. Albert R: **Scale-free networks in cell biology.** *J Cell Sci* 2005, **118**(Pt 21):4947–4957.

7. Huss M, Holme P: **Currency and commodity metabolites: their identification and relation to the modularity of metabolic networks**. *IET systems biology* 2007, **1**(5):280–5.

8. Ma H, Zeng A: **Reconstruction of metabolic networks from genome data and analysis of their global structure for various organisms**. *Bioinformatics* 2003, **19**(2):270–277.

9. Arita M: **The metabolic world of *Escherichia coli* is not small**. *Proc Natl Acad Sci USA* 2004, **101**(6):1543–1547.

10. Feist AM, Henry CS, Reed JL, Krummenacker M, Joyce AR, Karp PD, Broadbelt LJ, Hatzimanikatis V, Palsson BØ: **A genome-scale metabolic reconstruction for *Escherichia coli* K-12 MG1655 that accounts for 1260 ORFs and thermodynamic information**. *Mol Syst Biol* 2007, **3**:121.

11. Price ND, Reed JL, Palsson BØ: **Genome-scale models of microbial cells: evaluating the consequences of constraints**. *Nat Rev Microbiol* 2004, **2**(11):886–897.

12. Gama-Castro S, Jiménez-Jacinto V, Peralta-Gil M, Santos-Zavaleta A, Peñaloza-Spinola MI, Contreras-Moreira B, Segura-Salazar J, Muñiz-Rascado L, Martínez-Flores I, Salgado H, Bonavides-Martinez C, Abreu-Goodger C, Rodríguez-Penagos C, Miranda-Ríos J, Morett E, Merino E, Huerta AM, Treviño-Quintanilla L, Collado-Vides J: **RegulonDB (version 6.0): gene regulation model of *Escherichia coli* K-12 beyond transcription, active (experimental) annotated promoters and Textpresso navigation**. *Nucleic Acids Res* 2008, **36**(Database issue):D120–4.

13. Blot N, Mavathur R, Geertz M, Travers A, Muskhelishvili G: **Homeostatic regulation of supercoiling sensitivity coordinates transcription of the bacterial genome**. *EMBO Rep* 2006, **7**(7):710–5.

14. Saeed AI, Sharov V, White J, Li J, Liang W, Bhagabati N, Braisted J, Klapa M, Currier T, Thiagarajan M, Sturn A, Snuffin M, Rezantsev A, Popov D, Ryltsov A, Kostukovich E, Borisovsky I, Liu Z, Vinsavich A, Trush V, Quackenbush J: **TM4: a free, open-source system for microarray data management and analysis**. *BioTechniques* 2003, **34**(2):374–8.

15. Cleveland W, Devlin S: **Locally Weighted Regression: An Approach to Regression Analysis by Local Fitting**. *Journal of the American Statistical Association* 1988, **83**(403):596–610.

16. Pan W: **A comparative review of statistical methods for discovering differentially expressed genes in replicated microarray experiments**. *Bioinformatics* 2002, **18**(4):546–54.

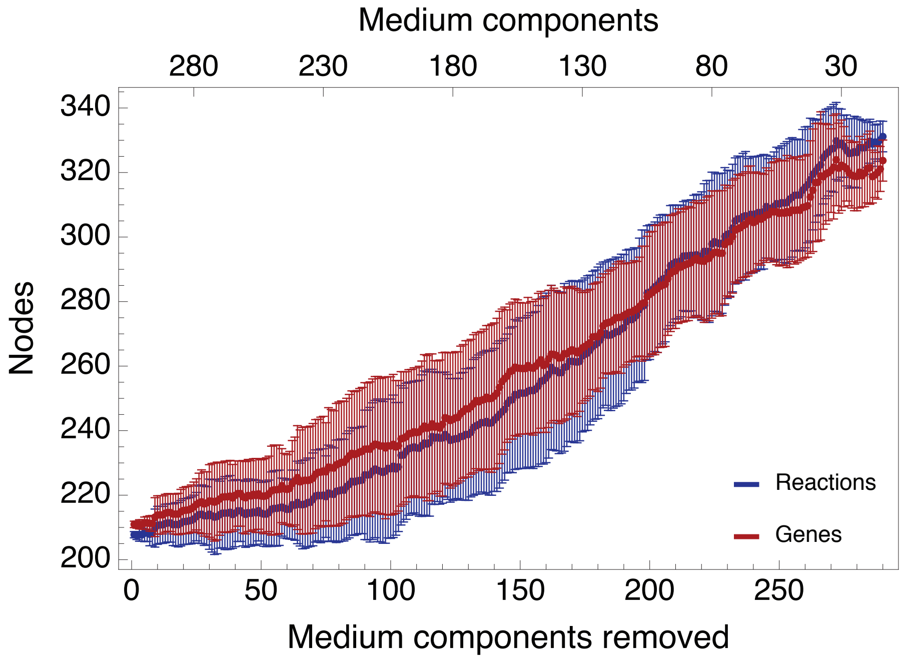

Figure S1. The number of active reactions and genes in the effective networks increases when moving from rich to minimal media conditions. Only cytosolic reactions and genes were counted (i.e. transport and periplasmic reaction were excluded).

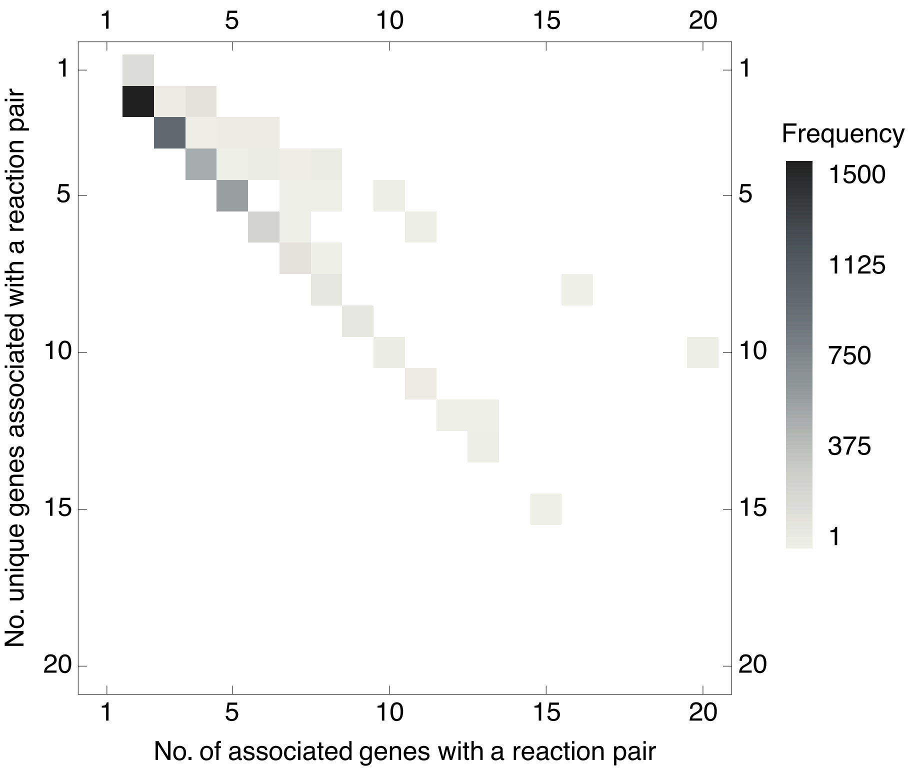

Figure S2. The multiplicities of reaction-gene relations depicted as a colored grid.

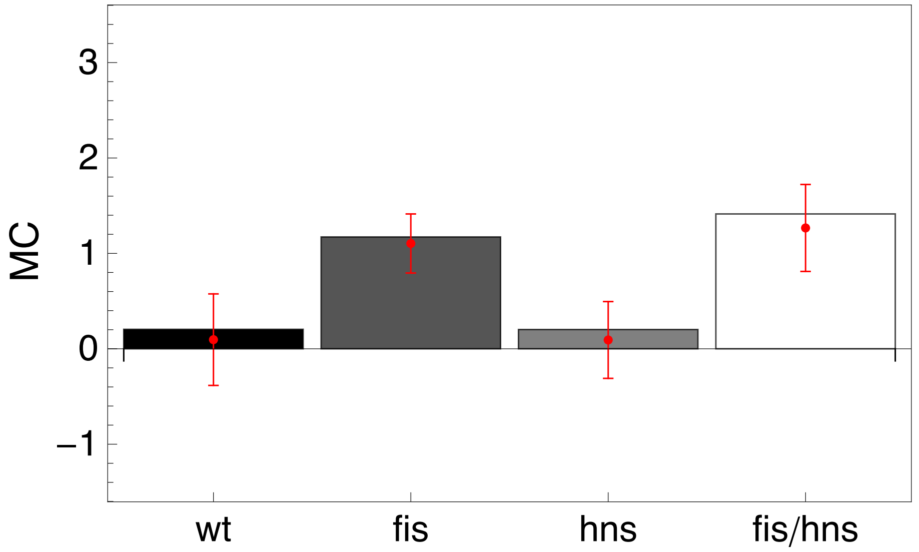

Figure S3. Effect of currency metabolites as seen for the untreated iAF1260 network

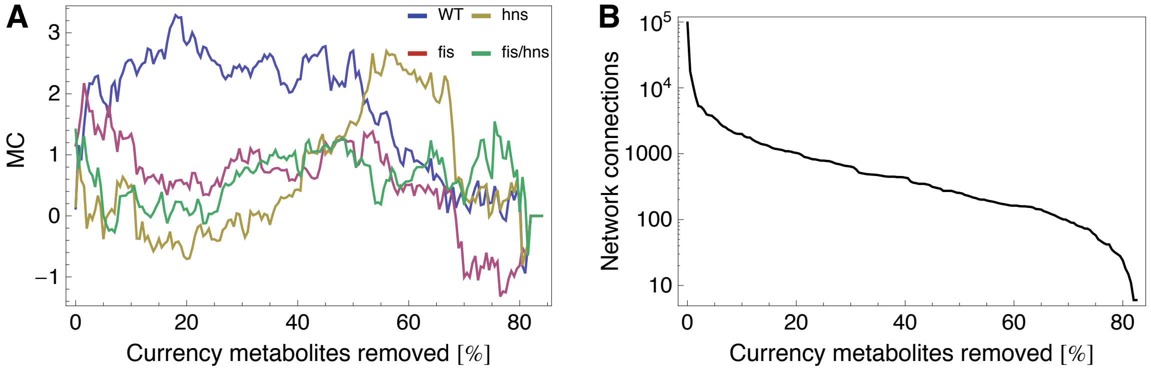

Figure S4. (A) MC values plotted against the percentage of removed currency metabolites (as determined by the degree threshold method). (B) The network connectivity, i.e. number of connections in the network, plotted against the percentage of removed metabolites.

Table S1. Table of the MC values visualized in Figure 5 (main article).

Label	Network	WT	*fis*	*hns*	*fis/hns*
1	EcoCyc	3.37	0.31	-0.60	-0.65
2	EcoCyc*	3.17	0.37	-0.53	-0.52
3	Intersection of EcoCyc and KEGG networks	3.03	0.26	0.54	-0.66
4	Intersection of EcoCyc and $iAF1260_{man}$	2.89	0.57	-0.40	-0.13
5	$iAF1260^{*}_{man}$	2.75	1.47	0.85	-0.05
6	$iAF1260_{man}$ obtained from FBA (rich medium)	2.69	1.21	-1.11	-0.38
7	$iAF1260_{man}$	2.62	1.61	0.85	-0.065
8	EcoCyc**	2.55	0.22	0.33	0.65
9	KEGG*	2.57	0.76	1.59	1.32
10	iAF1260*	2.50	1.51	0.03	0.55
11	$iAF1260_{deg}$	2.40	1.52	0.16	0.66
12	$iAF1260^{**}_{man}$	2.36	2.09	0.37	0.12
13	Flux-coupling network (fully coupled)	2.22	0.14	1.70	1.14
14	KEGG	2.20	0.61	1.31	1.40
15	Intersection of KEGG and $iAF1260_{man}$	1.80	1.43	-0.11	-0.46
16	Intersection of EcoCyc, KEGG and $iAF1260_{man}$	1.76	0.68	-0.26	-0.48
17	KEGG**	1.58	-0.17	-0.46	1.048
18	Flux-coupling network (fully and directionally coupled)	1.50	1.69	1.91	0.55
19	$iAF1260^{**}_{deg}$	1.37	1.62	0.14	-0.11
20	iAF1260**	1.20	1.17	0.42	1.17
21	Flux-coupling network (directionally coupled)	1.06	0.39	0.03	1.51
22	iAF1260	0.19	1.17	0.23	1.40
23	iAF1260*	0.11	1.14	0.13	1.41
